# Magnesium and Its Role in Primary Open Angle Glaucoma; A Novel Therapeutic?

**DOI:** 10.3389/fopht.2022.897128

**Published:** 2022-06-09

**Authors:** Mirna Elghobashy, Hannah C. Lamont, Alexander Morelli-Batters, Imran Masood, Lisa J. Hill

**Affiliations:** ^1^ School of Biomedical Sciences, Institute of Clinical Sciences, University of Birmingham, Birmingham, United Kingdom; ^2^ School of Chemical Engineering, Healthcare Technologies Institute, University of Birmingham, Birmingham, United Kingdom

**Keywords:** glaucoma, magnesium, oxidative stress, trabecular meshwork, retinal ganglion cells

## Abstract

Glaucoma is the leading cause of irreversible blindness globally, with Primary open angle glaucoma (POAG) being the commonest subtype. POAG is characterized by an increase in intraocular pressure (IOP), leading to optic nerve damage and subsequent visual field defects. Despite the clinical burden this disease poses, current therapies aim to reduce IOP rather than targeting the underling pathogenesis. Although the pathogenesis of POAG is complex, the culprit for this increase in IOP resides in the aqueous humour (AH) outflow pathway; the trabecular meshwork (TM) and Schlemm’s canal. Dysfunction in these tissues is due to inherent mitochondrial dysfunction, calcium influx sensitivity, increase in reactive oxygen species (ROS) production, TGFβ-2 induction, leading to a sustained inflammatory response. Magnesium is the second most common intracellular cation, and is a major co-factor in over 300 reactions, being highly conserved within energy-dependent organelles such as the mitochondria. Magnesium deficiency has been observed in POAG and is linked to inflammatory and fibrotic responses, as well as increased oxidative stress (OS). Magnesium supplementation been shown to reduce cellular ROS, alleviate mitochondrial dysregulation and has further antifibrotic and anti-inflammatory properties within ocular tissues, and other soft tissues prone to fibrosis, suggesting that magnesium can improve visual fields in patients with POAG. The link between magnesium deficiency and glaucoma pathogenesis as well as the potential role of magnesium supplementation in the management of patients with POAG will be explored within this review.

## Introduction

Glaucoma is the most common cause of irreversible blindness globally, affecting over 70 million people worldwide (1). This disease encompasses many conditions which contributes to progressive optic neuropathy, resulting from progressive retinal ganglion cell (RGC) degeneration and optic disc cupping (2). The commonest subtypes of glaucoma are; primary open angle glaucoma (POAG) and acute angle closure glaucoma (AACG), with POAG accounting for 70% of worldwide total glaucoma cases (3). Currently, the only modifiable risk factor, elevated intraocular pressure (IOP), contributes to this optic neuropathy and subsequent visual loss (4). The culprit for this increase in IOP lies within the anterior chamber of the eye, where aqueous humor (AH) outflow is regulated by the trabecular meshwork tissue (TM) (5). In POAG, the induction of a fibrotic response occurs within the TM, creating heightened resistance to fluid outflow, subsequently elevating IOP beyond normal levels (6). Furthermore, due to POAG having a multifactorial nature, vascular dysregulation and endothelial dysfunction within the Schlemm’s canal (SC), further contribute to the induction of a fibrotic response by increasing extracellular matrix (ECM) deposition, oxidative stress (OS), and apoptosis within the within the TM (7). With current therapies focusing on alleviating symptoms, such as lowering IOP, rather than targeting the underlying causes that contribute towards the pathogenesis of POAG (8), this has led to POAG bearing a high prevalence and posing a significant clinical burden (9). Therefore, it is important to consider alternative therapeutics that could be utilized to treat glaucoma.

Previous research has indicated that magnesium has the potential to be a promising therapeutic for fibrotic diseases due to its antioxidant and anti-fibrotic effects in the body within different organs (10, 11). In ocular tissues, magnesium is present mainly in the cornea, lens, retina and in the anterior chamber (12), with deficiency being linked to ionic and antioxidant imbalances (13). Furthermore, significant magnesium deficiency has been identified in patients with glaucoma (14, 15), with magnesium supplementation having been shown to improve the visual field of glaucoma patients (16).

This review will explore the link between POAG pathogenesis, and the role that magnesium could potentially play in the management of POAG focusing on its mechanism of action in mediating cellular homeostasis. As it is widely regarded that POAG originates in the anterior chamber of the eye, emphasis is placed on TM and vascular dysfunction occurring during AH outflow.

## Glaucoma Pathophysiology

Several mechanisms have been proposed for the pathogenesis of POAG, with TM fibrosis leading to impaired drainage of AH being widely regarded as a large contributor to this multi-faceted disease (17). Studies have identified a link between elevated TGF-β2 levels in the AH and extracellular matrix (ECM) of patients with POAG (18–21). It has been established that TGF-β isoforms regulate TM cellular fate due to their mesenchymal nature (22–24), with a heightened presence of TGF-β2 stimulating a myofibroblast phenotype, increasing contractile features, pro-fibrotic protein expression (α-SMA, fibronectin) and ECM deposition (25, 26). Overall, these factors contribute to a loss of cell-cell contact, increased stiffness, and fibrosis of the TM, leading to AH outflow resistance and increased IOP (26).

Being highly metabolizing cells, TM cells are also sensitive to overproduction of reactive oxidative species (ROS) (27). Increased production of ROS can be generated from cells within the anterior chamber of the eye from several organelles, such as; the mitochondria, endoplasmic reticulum, and cytosol, in response to exogenous stress such as UV damage, further leading to protein and cellular damage (28, 29). Similar events occur within the TM, with an imbalance in ROS production causing mitochondrial dysfunction, ECM accumulation, cytoskeletal changes and induction of apoptosis (28). This has been reflected in POAG patients, with a significant reduction in TM cellularity compared to age matched controls (30). Prolonged ROS production in POAG is also associated with inflammation and inflammatory stress to the TM (31). There is a release of proinflammatory cytokines such as IL-1β *via* the activation of the nuclear factor kappa b (NF-κB) pathway (32). NF-κB is a transcription factor which promotes genes related to inflammation to produce an inflammatory response (33). Chronic inflammation is pathognomonic of ocular hypertension, and the development of POAG (32, 34). Overall, these processes contribute to increased outflow resistance and thus increased IOP (35, 36).

## Magnesium and Its Role Within the Eye

Magnesium (Mg^2+^) is the fourth most abundant cation in the body, acting as a co-factor in over 300 enzymatic reactions (37), particularly those required in ATP-generating reactions and regulating mitochondrial functions (13, 38). Intracellular magnesium concentrations remain virtually unaltered due to tight intracellular regulation, despite significant gradients across cell membranes and being the second most abundant intracellular cation. This gives appreciation into how tightly regulated magnesium intracellular stores are, with the mitochondria, endoplasmic reticulum and nucleus holding the largest stores of magnesium, with around 15-18mM magnesium present in each organelle (39–41). While it has been noted in studies that there are several conserved magnesium co-transport mechanisms across cell membranes, there are also compensatory mechanisms that allow the cell to effectively buffer any loss or overaccumulation of this cation (41, 42). With magnesium having a high impact on energy metabolism, inevitably effecting cellular responses (13), it is also considered a potent antagonist against the stimulatory effects of calcium, an important cation associated with the implications of POAG (13, 43). Thus, what should be considered in future studies is how magnesium can directly and indirectly effect POAG patients and what mechanism(s) of action will be affected for maintaining homeostasis and normal cellular functions.

Within the eye, magnesium is present in high concentrations within the cornea, lens, retina and the anterior chamber (12). In the cornea, magnesium is shown to be essential in preventing dry eye disease and infection (44) and acts as a neuroprotective and anti-apoptotic agent by reducing nitric oxide synthase and the induction of calcium channels in RGCs (15, 45, 46). Furthermore, as TM cells are highly metabolic and can possess a smooth muscle phenotype, the cells express several known transporters that are known to mediate the tissues contractility by enhancing intracellular calcium stores, hence mediating fluid outflow (47, 48). Thus, with magnesium being essential in cellular metabolism, and regulation of intracellular anionic balance, it is likely to be vital for the maintenance of ocular structural and cellular integrity (15). This is further evident in the case of magnesium concentrations in the AH of healthy patients being estimated at 6.7mg/L, compared to patients with POAG, at around 3mg/L (14). While current studies into magnesium levels in POAG patients is limited, it is worth highlighting that a potential relationship could be correlated from the existing literature. The current evidence emphasizes the importance of physiological magnesium levels for normal ocular tissue function, further suggesting that research into how magnesium can directly and indirectly manage POAG through various mechanisms of action could be explored for therapeutic potential.

**Figure 1:**
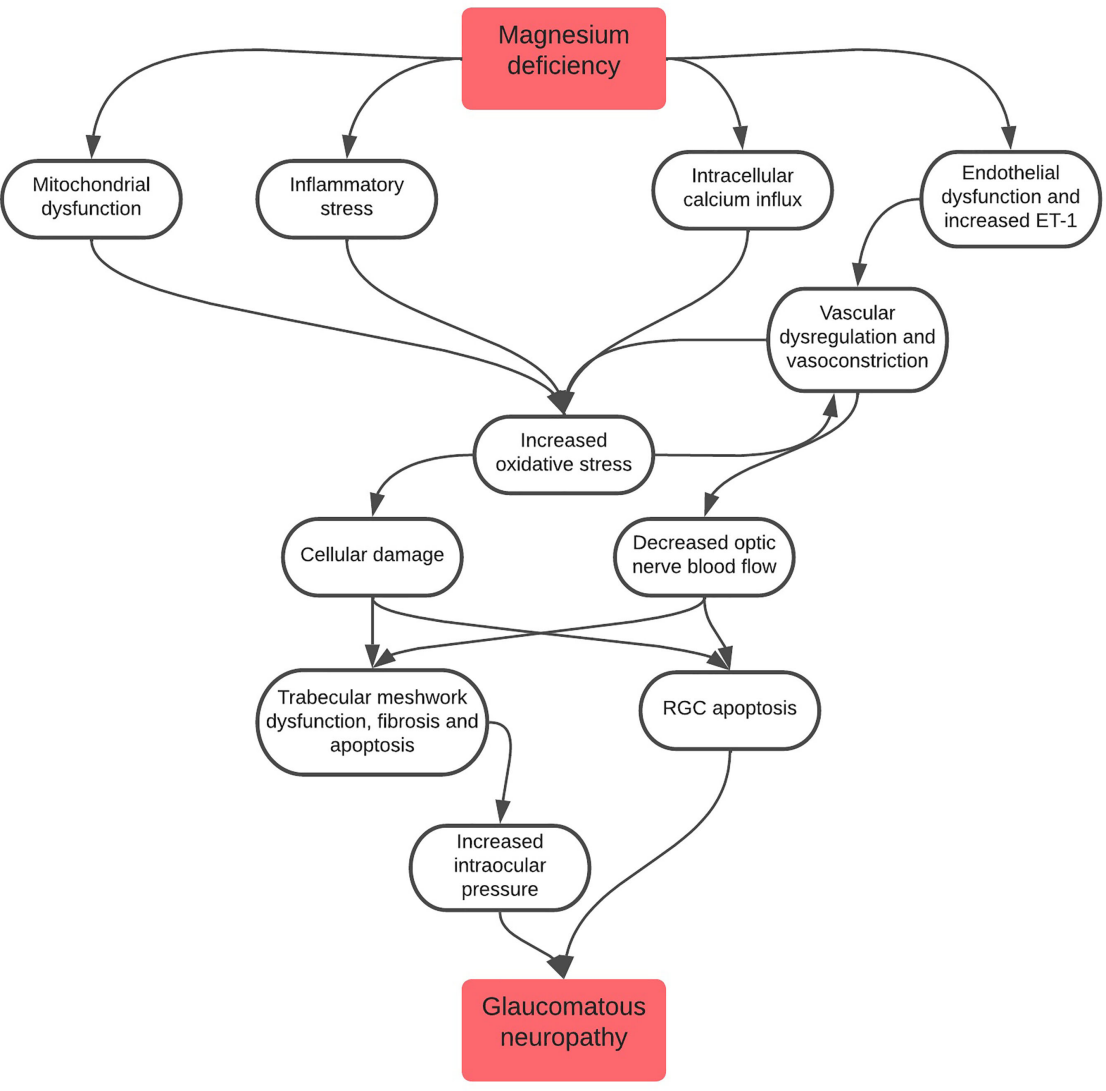
Flowchart depicting the role of magnesium deficiency in the pathogenesis of glaucomatous neuropathy. It is important to note that all pathways lead to increased oxidative stress, a major driver of glaucomatous neuropathy. Oxidative stress also leads to a cycle of increased vascular dysregulation and vasoconstriction which, in turn, causes further oxidative stress to cells.

This review will discuss several roles in which magnesium deficiency is linked to POAG pathogenesis (**Figure 1**), with major outcomes of this deficiency consisting of mitochondrial dysfunction, inflammatory stress and endothelial dysfunction.

## Role of Magnesium in Oxidative Stress and Ocular Pathologies

Oxidative stress (OS) is defined as an imbalance of oxidants to antioxidants, leading to lipid peroxidation and protein damage, further perpetuating an ongoing cycle of OS, causing aberrant ECM deposition, cytoskeletal changes within TM cell, and eventual apoptosis (49). The majority of intracellular ROS are generated in the mitochondria, as a by-product of cellular respiration (50), with the accumulation of intracellular ROS production being influenced by mitochondrial functionality. Likewise, magnesium deficiency can also affect mitochondrial function due to being a major co-factor necessary in energy production (13). This holds true within the eye, as OS is associated with the pathogenesis of several ocular pathologies; cataracts, retinopathies, age-related macular degeneration and glaucoma (27). Specifically, within POAG, it is widely regarded that mitochondrial dysfunction and genetic abnormalities are associated with complex I of the electron transport chain and are common attributes found within patients (51, 52). It is hypothesized that it is this defect in the electron transport chain that contributes to the progressive loss of TM cells in POAG, abnormal ATP production, increased ROS production and heightened vulnerability to calcium ion stress (52, 53). It has previously proposed that the introduction of antioxidants may help subside these effects and decrease IOP (54, 55).

Specifically, magnesium deficiency disrupts the electron transport chain within the mitochondria by facilitating the uncoupling of oxidative phosphorylation leading to electron loss (56, 57). As previously mentioned, magnesium is vital with regards to ATP and its production. *In vitro* studies have identified that many magnesium-dependent enzymes and dehydrogenases are necessary in the mitochondria to maintain cellular homeostasis, with computer simulation models of the Krebs cycle showing that mitochondrial magnesium levels are essential for the regulation of these enzymes and the Krebs cycle itself (13, 58, 59), indicating that magnesium is a vital regulator of mitochondrial homeostasis and, by extension, metabolic status. The known magnesium deficiency in patients with POAG could be contributing to mitochondrial dysfunction within various ocular cell types, giving rise to ROS, calcium ion sensitivity and subsequent tissue impairment (14, 58). Current evidence illustrates the importance of conserving magnesium in the mitochondria, with deficiency indirectly influencing OS through the induction of stress responses by effecting the calcium (Ca^2+^) intracellular homeostasis (60). It has been observed that magnesium deficiency leads to increased intracellular calcium influx and thus a decreased ratio of magnesium to calcium (11). Increased intracellular calcium causes a myriad of cellular imbalances, inducing intracellular swelling and apoptosis, with this dysfunction observed in POAG TM cells (43, 61). This is also important when considering the RGC loss observed in glaucoma, leading to visual defects (61, 62).

While magnesium deficiency has been associated with POAG, a direct link between magnesium deficiency and increased OS in the eyes has not yet been fully elucidated (14). The impact of magnesium related modulation of OS has been explored through the use of rodent models; magnesium deficient rats are more susceptible to oxidative damage than control rats and have higher levels of circulating oxidized lipoproteins (63). Furthermore, in a rodent glaucoma model, magnesium acetyltaurate has been shown to reduce OS and reduce RGC apoptosis (64). While these studies have created a foundation in forming a link between the presence of magnesium of OS levels, further understanding its therapeutic potential in minimizing ROS production and overall cellular health in the TM samples of patients still remains to be elucidated.

## Magnesium and Vascular Regulation

Several studies have suggested a link between magnesium deficiency and vascular dysfunction (65–67), with general dysfunction of the microvascular endothelium being identified as a contributing factor of glaucomatous optic neuropathy (66). Studies have shown that blood flow is reduced in several ocular tissues in patients with glaucoma including the retina, optic nerve and choroid due to systematic hypertension (68, 69). This can further cause ocular endothelial damage and ocular hypertension leading to dysfunctional TM and hence increase IOP in POAG (70–72).

POAG has been associated with primary vascular dysregulation, wherein ocular and retinal vascular tissue responds abnormally to stimuli, due to low blood pressure and disturbed vascular homeostasis, causing abnormal AH outflow (73). Moreover, decreased available blood flow leads to an hypoxia-induced increase in OS within these tissues (74). These scenarios have shown to cause a marked increase in endothelin-1 (ET-1), a potent vasoconstrictor dependent on voltage-gated calcium channels to exert its actions. This can be potentially modulated by magnesium, which has been observed to attenuate such events that may arise from aberrant calcium homeostasis (75–77). It has also been theorized that ET-1 acts on the distal vascular post-TM and SC to increase resistance (78, 79), as activation of ET-1 receptors in the TM induces tissue contraction, increasing fluid outflow resistance, and raises IOP. Magnesium has been identified in *ex vivo* studies as a substance which reduces the vasoconstrictive effect of ET-1 and inhibiting ET-1 vasoconstriction in porcine ciliary arteries (80, 81). Although the evidence for the use of magnesium as a preventative vasorelaxant is largely theoretical when discussing ocular vasculature, there is a significant correlation between magnesium’s role as a physiological calcium channel blocker and its action in ET-1 inhibition and has been shown to be beneficial for chronic hypertension (15, 77, 80).

Magnesium deficiency is also associated with endothelial dysfunction (82). Normally, the endothelium of ocular blood vessels produces signaling molecules which control the balance between thrombin and fibrinolysis as well as the synthesis of proinflammatory cytokines and reduction of nitric oxide (11, 83, 84). It has been suggested that paracrine signaling between the TM and SC regulates vascular tone and is similar to that found in vascular smooth muscle endothelium, in the presence of ET-1 and nitric oxide (85). Moreover, in magnesium deficiency, it has been shown there is increased intracellular nitric oxide and peroxynitrite production, giving rise to an increased levels of ROS (86, 87). A study assessing the use of magnesium on glaucoma patients as a ‘physiological calcium channel blocker’, found that supplementation led to significant improvements in peripheral circulation and visual fields (16). Although further studies have not been conducted assessing the validity of this finding, it adds weight to the argument that supplementation with magnesium could induce vasodilation and reduce the vasoconstrictor effects of ROS in glaucoma patients (88).

## Role of Magnesium in Inflammation and Fibrosis

Magnesium deficiency has been linked with inflammation in the context of chronic disease and it has been theorized that the development of POAG, as TM dysfunction and ocular hypertension is mediated by chronic low-level inflammation (32, 34, 89). It has been suggested that there is a release of proinflammatory cytokines such as IL-1β *via* the activation of the NF-κB pathway in patients with POAG (32, 90). NF- κB being a primary transcription factor which promotes genes related to inflammation, has an intimate cross-talk with the TGFβ-2 signaling pathway (33, 91, 92). Studies have identified elevated TGF-β2 levels in the AH of POAG patients, with a heightened presence associated with the induction of ROS, and overproduction of profibrotic proteins in TM cells (18, 91, 93, 94). Furthermore, TGF-β2 has been shown to contribute to the myofibroblastic properties and increased ECM deposition seen in glaucomatous TM (95). Although there is no evidence linking the specific role of magnesium in TM fibrosis, it may have the potential to reduce fibrosis indirectly in the TM through alternative pathways previously discussed. As magnesium is a primary mediator in metabolic processes and mitochondrial functionality, the effects this cation has on ROS production, could be beneficial in reducing TGFβ-2 production, as it has been previously shown that the presence of mitochondrial targeted antioxidants has the potential to attenuate TGFβ-2 in TM cells (96). This may in turn, have an indirect, and advantageous effect on the inflammatory response that is induced through the NF-κB pathway.

As magnesium deficiency is common in POAG patients, it can also be observed in other fibrotic diseases, with supplementation currently being trialed in soft tissues, such as liver and lungs (10, 97). In a rat model, magnesium deficiency led to development of systemic inflammatory disease, and magnesium supplementation resulted in decreased inflammatory and immunological responses (98). Moreover, in a trial of middle-aged women, lower serum magnesium levels were associated with a significantly raised C-reactive protein (CRP), an acute phase protein which is a valuable marker for inflammatory stress (99). It was also found that short-term magnesium supplementation reduced inflammatory cytokine production in both mothers and neonates, including a reduction in NF-κB and IL-6 (100). This adds weight to the argument that magnesium supplementation can also be beneficial for POAG patients as anti-inflammatory agent although it is yet to be seen whether the effects observed would also be seen in the eye.

In terms of fibrosis, hepatic fibrosis has a similar pathogenesis to POAG including excess ECM accumulation, as hepatocytes are also highly metabolic cells (101–103). Insults to the liver stimulate hepatic stellate cells to become pro-fibrotic and adopt myofibroblastic properties *via* the NF-κB pathway, alike the TM (101, 104). Paik et al. (2011) has shown that magnesium supplementation in rats with hepatic fibrosis reduced α-SMA, TGF-β and collagen expression, all profibrotic proteins overexpressed by TM cells during POAG pathogenesis (10, 105). Additionally, ROS generation was suppressed, and transcriptional activation of NF-κB was reduced (10). Similar findings with magnesium supplementation has been shown to alleviate pulmonary fibrosis in mouse models through similar pathways related to POAG (97, 106). It was later indicated by Luo et al. (2021) that magnesium inhibited TGF-β-induced myofibroblastic changes in primary lung cells and decreased collagen deposition in human lung fibroblasts *via* regulation of the TGF-β/SMAD pathway (97). These findings suggest that magnesium has an influence in mediating an anti-fibrotic effect that is similar to the fibrotic pathogenesis seen in the TM and may show promise as a potential anti-fibrotic treatment.

## Conclusion

As glaucoma is a multi-factorial disease, there are several pathways that have been considered that contribute towards disease pathogenesis, such as inherent mitochondrial dysfunction, subsequent OS stress, calcium influx sensitivity, pro-fibrotic protein induction and vascular dysregulation. It has been presented within this review that the functionality of these same pathways have been shown to be restored with magnesium supplementation in POAG and other fibrotic disease types such as hepatic and pulmonary fibrosis. This suggests that magnesium could be potentially promising in its applications for reducing POAG symptoms by reducing oxidative damage, vascular dysfunction, inflammation and fibrosis, alike other fibrotic diseases.

Overall, it is established that magnesium is vitally important in the regulation and homeostasis of ocular tissues by maintaining cellular functionality. The association of magnesium deficiency in POAG patients and the pathogenesis of glaucoma potentially attributed to magnesium’s role as an important cofactor in enzymatic reactions, metabolic and OS regulation, all associated with TM dysregulation, POAG and AH fluid outflow. It has not yet been fully determined the exact impact of magnesium deficiency on the development and progression of glaucoma, however it should be emphasized that there is room in the field for further investigation into its beneficial effects.

## Author Contributions

ME and HL; preparation of original draft and review of the manuscript. AM-B, IM, and LH; edited and reviewed the manuscript. All authors contributed to the article and approved the submitted version.

## Funding

This work was supported by EPSRC and SFI Centre for Doctoral Training in Engineered Tissues for Discovery, Industry and Medicine (Grant number EP/S02347X/1).

## Conflict of Interest

The authors declare that the research was conducted in the absence of any commercial or financial relationships that could be construed as a potential conflict of interest.

## Publisher’s Note

All claims expressed in this article are solely those of the authors and do not necessarily represent those of their affiliated organizations, or those of the publisher, the editors and the reviewers. Any product that may be evaluated in this article, or claim that may be made by its manufacturer, is not guaranteed or endorsed by the publisher.
